# Urine albumin-to-creatinine ratio diurnal variation rate predicts outcomes in idiopathic membranous nephropathy

**DOI:** 10.1007/s10157-023-02444-9

**Published:** 2024-01-19

**Authors:** Xiaoqing Chen, Yong Zhang, Liqun Yan, Yangbin Xie, Shujing Li, Yongze Zhuang, Liping Wang

**Affiliations:** 1 Fuzong Clinical Medical College of Fujian Medical University, Fuzhou, 350025 China; 2 Department of Nephrology, The 900th Hospital of Joint Logistics Support Force, 156 West Second Ring Road, Fuzhou, 350025 People’s Republic of China

**Keywords:** Albumin-to-creatinine ratio, Circadian rhythm, Diurnal variation, Idiopathic membranous nephropathy, Proteinuria, Remission

## Abstract

**Background:**

Idiopathic membranous nephropathy (IMN) is a leading cause of end-stage renal disease (ESRD). The purpose of this study was to evaluate whether urinary albumin-to-creatinine ratio (UACR) diurnal variation rate calculated by spot urinary protein test predicts 1-year nephrotic outcomes as a biomarker of proteinuria severity in patients with IMN.

**Methods:**

Patients’ baseline demographics, blood and urinary biomarkers, and clinical and pathological characteristics were collected retrospectively. Urine samples were collected at 7:00 (before breakfast) and 19:00 (after dinner) to calculate the UACR diurnal variation rate. A prediction model for no remission (NR) was developed statistically based on differences between prognosis groups. Receiver operating characteristic curve (ROC) analysis was performed to evaluate prediction abilities and determine optimal cut-off points of the model and UACR diurnal variation rate alone.

**Results:**

The formula for calculating the probability of NR was exp(L)/(1 + exp(L)), where the linear predictor L =  – 22.038 + 0.134 × Age (years) + 0.457 × 24-h urinary protein + 0.511 × blood urea nitrogen (BUN) + 0.014 × serum uric acid (SUA) + 2.411 if glomerular sclerosis + 0.816 × fasting blood glucose (FBG)-0.039 × UACR diurnal variation rate (%). Optimal cut-off points for NR prediction by the final model and UACR diurnal variation rate alone were 0.331 and 58.5%, respectively. Sensitivity and specificity were 0.889 and 0.859 for the final model, and 0.926 and 0.676 for UACR diurnal variation rate alone.

**Conclusion:**

UACR diurnal variation using spot urinary protein is a simpler way to predict nephrotic outcomes and is a highly sensitive screening tool for identifying patients who should undergo further comprehensive risk assessment.

**Supplementary Information:**

The online version contains supplementary material available at 10.1007/s10157-023-02444-9.

## Introduction

Membranous nephropathy (MN) is the most common type of glomerulonephritis in adults, and 75% of patients are diagnosed as idiopathic membranous nephropathy (IMN), indicating that glomerular damage caused by an autoimmune response. The typical presentation of IMN is nephrotic syndrome with preserved renal function, which is shown in 80% of patients [1].The pathogenesis starts with the deposition of immune complex outside the glomerular basement membrane (GBM), which further activates complement and finally leads to structural damage of GBM, represented by proteinuria [2]. In the natural history of IMN, 30–35% of patients achieve spontaneous remission; however, 20–40% of patients may eventually progress to end-stage renal disease (ESRD) within 5–10 years, characterized by poorer quality of life and shortened life expectancy [3, 4].

The rate of spontaneous remission varies in patients with different grades of proteinuria; a more advanced proteinuria grade is associated with a higher risk of progression to ESRD. Thus, the goal of IMN treatment is to reduce proteinuria severity as long as possible. Complete remission (CR) of nephrotic syndrome predicts both long-term patient and renal survival, and partial remission (PR) significantly reduces the risk of ESRD. The reduction in proteinuria is noticeably slow for years in some patients, and 30–40% of patients with persistent nephrotic syndrome may develop ESRD over 10 years [5]. Thus, because of the long-lasting and varied nature of IMN, routine assessment of the degree of proteinuria is critical in order to understand disease progression and treatment response. In addition, clinical decision-making for the selection of proper treatment for IMN must always consider the benefit–risk ratio, since all available options show significant toxicity and adverse events, including severe infection due to immunosuppression and the link between some agents and higher malignancy risk [6, 7]. Identifying high-risk patients correctly for treatment and monitoring treatment efficacy aiming to minimize unnecessary exposure is essential.

The gold standard for determining the degree of proteinuria is assaying 24-h urinary protein excretion. However, the collection process is time-consuming and cumbersome, so usually spot urinary protein or spot urinary albumin-to-creatinine ratio (UACR), in which protein levels readjusted by creatine, is used as a substitute test. Whether spot urinary protein or spot UACR can completely represent the true urinary protein excretion, is a hot topic of discussion and research in this field, and discrepancies between results have been reported in numerous studies of several renal diseases. For example, Hogan et al. reported that the correlation between spot urinary protein and 24-h urinary protein in patients with biopsy-proven glomerular diseases was poor[8]. Finding new biomarkers or new adjustment methods for existing markers, such as UACR, for a more accurate evaluation of proteinuria may help to improve IMN management. Circadian rhythms are defined as behavior and physiology cycles that are synchronized to the day/night cycle, which has been observed in several renal processes, including glomerular filtration rate (GFR), renal plasma flow, and excretion of water and urinary solutes [9–11]. Considering these possible effects, the present study aimed to evaluate UACR diurnal variation using spot urinary protein to determine whether a simpler assessment of proteinuria level is able to predict nephrotic outcomes.

## Methods

### Patients

Patients with IMN who were diagnosed according to the Kidney Disease: Improving Global Outcomes (KDIGO) 2012 recommendations [6] in our hospital between January 2015 and June 2021 were screened for eligibility. Inclusion criteria were patients aged between 18 and 70 years, with an estimated glomerular filtration rate (eGFR) ≥ 60 ml/min/1.73 m^2^[12], naïve to glucocorticoid and/or immunosuppressant treatments at baseline, with sufficient clinical records to assess, and at least 1-year follow-up or until the time to reach CR or composite renal outcome within 1 year. Patients with secondary membranous nephropathy (SMN), combined with other types of glomerulonephritis, combined with severe infection, cardiovascular, hepatic, or neurological diseases, or pregnancy were excluded. Patients were followed every 2 weeks until stable, then followed monthly or bimonthly as decided by the nephrologists. The treatment during the study period were decided by the nephrologists according to KDIGO 2012 recommendations [6].

### Ethical considerations

This study was reviewed and approved by the ethics committee of the Institutional Review Board of our hospital (Number: 2011-011), and signed informed consent was provided by all included patients.

### Variables

Demographic data, including age and sex, and clinical data, including body mass index (BMI), systolic blood pressure (SBP), diastolic blood pressure (DBP), smoking and drinking status, and comorbidities, were collected at baseline. Medications used during the study period were recorded. Blood samples were collected for measurement of albumin, serum creatinine (SCr), blood urea nitrogen (BUN), serum uric acid (SUA), fasting blood glucose (FBG), alanine aminotransferase (ALT), aspartate aminotransferase (AST), total cholesterol (TC), triglyceride (TG), low-density lipoprotein cholesterol (LDL-C), high-density lipoprotein cholesterol (HDL-C), hemoglobin, platelets and immunoglobulin (IgG, IgA, and IgM), complement C3 and C4, D-dimer, fibrinogen, and phospholipase A2 receptor (PLA2R) IgG. PLA2R IgG was measured by indirect immunofluorescence assay (IFA, Euroimmun AG, Lübeck, Germany). Albumin, SCr, and BUN levels were measured at baseline and each follow-up visit, while the others were measured once at baseline.

### Urine testing

Urine spot test and 24-h urinary protein, albumin, and creatine levels were assayed at baseline and each follow-up visit using the Cobas 8000 c702 Analyzer (Roche Diagnostics, Mannheim, Germany). Visual and microscopic examinations were performed to check abnormalities, including the presence of blood, infections, or crystals. For measurement and calculation of the UACR diurnal variation rate, urine samples were collected at 7:00 (before breakfast) and 19:00 (after dinner) for each patient continuously for 2 days, and calculated as (UACR_19:00_-UACR_7:00_)/UACR_7:00_, with average values for the consecutive 2 days used for analysis. For minimizing the possible bias related to physical activity, patients were hospitalized at least 1 day and the physical activity was monitored and restricted within 0.525 kcal/kg body weight or 3 METs for 10 min.

### Pathological evaluation

Biopsied renal tissue was evaluated at baseline by certified pathologists who were blinded to patients’ diagnoses and clinical outcomes. Immunohistopathological, light- and electron-microscopy examinations were conducted. Higher Ehrenreich–Churg staging was chosen when two stages were identified in specimens from the same patient [13]. The percentages of pathological glomeruli, including glomerular sclerosis or segmental glomerular sclerosis, percentage of the affected area of tubular atrophy or interstitial fibrosis, and mesangial proliferation grading were determined[14].

### Outcomes measurement

Clinical outcomes were evaluated according to the KDIGO 2012 recommendations at 1 year after IMN presentation [6]. In brief, CR was defined as spot urinary protein < 0.3 g/d or urine protein: creatinine ratio (uPCR) < 30 mg/mmol by two values at least 1 week apart, with normal blood albumin and SCr levels, and remission of nephrotic syndrome. Partial remission (PR) was defined as spot urinary protein > 0.3 to < 3.5 g/d or uPCR > 30 to < 350 mg/mmol and ≥ 50% reduction by two values at least 1 week apart, improved or reached normal blood albumin and SCr, and remission of nephrotic syndrome. In no remission (NR), urine and blood indexes do not fit the above criteria, and nephrotic syndrome persists. Nephrotic syndrome is defined by heavy proteinuria and hypoalbuminemia [15]. Composite renal outcome is defined as ≥ 30% reduction of eGFR, doubling of SCr from baseline, or ESRD. Patients who did not reach the end point were followed until March 1, 2022.

### Statistical analysis

Statistical analyses were assessed using the software IBM SPSS Statistics 25.0 (IBM Corporation, Armonk, New York, USA). All statistical hypothesis tests were two-sided with a significance level of 0.05. Continuous data are presented as mean with standard deviation (mean ± SD), and differences between prognosis groups (CR, PR, NR) were tested by one-way analysis of variance (ANOVA). Post hoc tests for the comparison of each two groups were performed using Bonferroni correction. Data with skewed distributions are presented as median with inter-quartile range (IQR, range between the 25th and 75th percentiles) and the differences between groups were tested using the non-parametric Kruskal–Wallis test, and comparisons between each two groups were performed by non-parametric Mann–Whiney test with Bonferroni correction. Categorical data are presented by count and percentage and Fisher’s exact test was performed to test their associations with prognosis. Comparison between each two groups were tested with two-sample Z-test for proportions with Bonferroni correction. Univariable and multivariable logistic regression models were constructed to find the independent predictors for NR. Variables in the univariable logistic regression model with *p* value < 0.2 were considered as possible independent predictors and are presented by odds ratios (ORs) with 95% confidence intervals (CIs). Possible predictors were then entered into multivariable analysis, with variables stepwise excluded from the multivariable model according to the backward conditional method. The final model presents adjusted ORs with 95% CIs of predictors, and a formula for estimating the probability of NR was established. Receiver operating characteristic curve (ROC) analysis was performed to evaluate the prediction ability. The optimal cutoff points were determined as the points with maximum Youden’s index. The corresponding sensitivity, specificity, positive predictive value (PPV), negative predictive value (NPV), and accuracy were predicted. Kaplan–Meier survival analysis was performed to show the cumulative renal survival rate and the rates of CR or PR, for the two groups stratified by UACR diurnal variation rate. Log-rank test was used to test differences in the cumulative rates between the two groups of patients.

## Results

### Demographic and clinical characteristics

A total of 439 patients were enrolled: 98 aged under 18 years or with SMN, 29 aged over 70 years, 117 had experienced glucocorticoid and/or immunosuppressant treatments, 30 had acute renal injury or eGFR < 60 ml/min/1.73 m^2^, 13 had incomplete medical records, 41 without data of pathological evaluation, 4 refused to participate, thus 107 patients remained. 9 patients were lost of follow-up, finally 98 patients were enrolled (*N* = 52 in the CR, 19 in the PR, and 27 in the NR groups) for analysis. Patients in the NR group were significantly older than those in the CR (mean ages 56.44 vs. 48.94 years, *P* < 0.05) and PR groups (mean ages 56.44 vs. 47.37 years, *P* < 0.05). The proportion of alcohol consumption in the PR group was significantly higher than that in the CR group (42.1% vs. 13.5%, *P* < 0.05). The proportion of patients with DM in the PR group was significantly higher than that in the CR group (36.8% vs. 11.5%, *P* < 0.05). Patients in the NR group had significantly higher 24-h urinary protein (medians of 4.9 vs. 3.8 g/day, *P* < 0.05) and BUN (medians of 6.38 vs. 4.51 mmol/L, *P* < 0.05) levels than those in the CR group. No significant differences were found in other demographic and laboratory values between the groups. The proportion of antiplatelet use in the PR group was significantly higher than that in the CR group (68.4% vs. 26.9%, *P* = 0.004). No significant differences were found in the use of other medications between groups (Table 1).

**Table 1:** Patients’ demographic and clinical data

	Total (*N* = 98)	CR (*N* = 52)	PR (*N* = 19)	NR (*N* = 27)	*P*-value
*Demographics*					
Age (years)^1^	50.70 (10.73)	48.94 (11.97)	47.37 (9.79)	56.44 (5.79)†‡	0.003*
Gender^3^					
Female	28 (28.6%)	18 (34.6%)	3 (15.8%)	7 (25.9%)	0.300
Male	70 (71.4%)	34 (65.4%)	16 (84.2%)	20 (74.1%)	−
BMI (kg/m^2^)^1^	24.16 (2.82)	23.90 (2.64)	24.27 (2.63)	24.57 (3.29)	0.605
SBP (mmHg)^1^	130.32 (17.69)	127.77 (19.05)	132.11 (13.07)	133.96 (17.56)	0.301
DBP (mmHg)^1^	84.11 (10.05)	82.62 (10.94)	86.26 (8.25)	85.48 (9.25)	0.286
Smoking^3^	28 (28.6%)	12 (23.1%)	7 (36.8%)	9 (33.3%)	0.456
Drinking^3^	19 (19.4%)	7 (13.5%)	8 (42.1%)^†^	4 (14.8%)^‡^	0.034*
*Comorbidities*					
Hypertension^3^	48 (49.0%)	19 (36.5%)	12 (63.2%)	17 (63.0%)	0.037*
DM^3^	21 (21.4%)	6 (11.5%)	7 (36.8%)†	8 (29.6%)	0.030*
Nephrotic syndrome^3^	81 (82.7%)	40 (76.9%)	16 (84.2%)	25 (92.6%)	0.267
Hematuria^3^	54 (55.1%)	31 (59.6%)	10 (52.6%)	13 (48.1%)	0.610
*Laboratory examinations*					
24-h urinary protein (g/day)^2^	4.4 (3.4, 6.1)	3.8 (3.1, 5.1)	5.0 (2.4, 7.1)	4.9 (3.8, 7.8)^†^	0.018*
Albumin^1^ (g/L)	25.65 (6.36)	26.67 (5.33)	24.44 (9.08)	24.56 (5.82)	0.247
SCr^1^ (μmol/L)	75.30 (14.70)	72.73 (14.31)	76.23 (15.00)	79.58 (14.69)	0.138
BUN^1^ (mmol/L)	5.16 (1.98)	4.51 (1.10)	5.20 (2.02)	6.38 (2.64)†	< 0.001*
SUA^1^ (μmol/L)	362.64 (93.56)	346.57 (89.31)	362.56 (77.21)	393.66 (106.61)	0.105
FBG^1^ (mmol/L)	5.37 (1.41)	5.16 (0.99)	5.50 (1.11)	5.70 (2.10)	0.257
ALT^2^ (U/L)	16.8 (11.9, 25.0)	16.2 (11.9, 25.5)	16.3 (12.1, 23.7)	18.8 (10.7, 30.9)	0.874
AST^2^ (U/L)	19.0 (15.7, 27.0)	19.1 (15.5, 27.0)	18.6 (15.7, 21.8)	19.4 (16.0, 28.1)	0.725
TC^1^ (mmol/L)	8.69 (2.95)	8.33 (2.48)	9.03 (3.96)	9.14 (2.99)	0.443
TG^2^ (mmol/L)	2.6 (1.6, 3.9)	2.3 (1.6, 3.3)	3.8 (1.6, 4.1)	2.9 (2.2, 4.6)	0.063
LDL-C^1^ (mmol/L)	5.96 (2.42)	5.97 (2.46)	6.15 (2.74)	5.82 (2.18)	0.906
HDL-C^1^ (mmol/L)	1.45 (0.46)	1.49 (0.47)	1.33 (0.41)	1.48 (0.47)	0.412
Hemoglobin^1^ (g/L)	134.83 (18.42)	135.44 (15.71)	137.42 (22.36)	131.81 (20.52)	0.566
Platelets^1^ (× 10^9^/L)	270.78 (59.46)	281.94 (53.03)	263.05 (86.89)	254.70 (43.41)	0.127
IgG^2^ (g/L)	5.5 (4.2, 7.0)	5.7 (4.6, 7.3)	5.9 (3.8, 8.6)	5.4 (4.1, 6.9)	0.582
IgA^2^ (g/L)	2.0 (1.6, 2.6)	1.9 (1.6, 2.4)	2.1 (1.5, 3.2)	2.0 (1.5, 2.3)	0.457
IgM^2^ (g/L)	1.0 (0.7, 1.4)	1.0 (0.7, 1.4)	0.9 (0.7, 1.6)	1.0 (0.7, 1.5)	0.990
Complement C3^1^ (g/L)	1.15 (0.25)	1.14 (0.21)	1.18 (0.34)	1.14 (0.26)	0.796
Complement C4^1^ (g/L)	0.27 (0.09)	0.27 (0.09)	0.28 (0.09)	0.26 (0.09)	0.633
D-dimer^2^	0.7 (0.4, 2.0)	0.7 (0.5, 1.7)	0.8 (0.5, 3.4)	0.6 (0.3, 2.7)	0.844
Fibrinogen^1^ (g/L)	4.71 (1.28)	4.74 (1.36)	4.72 (1.19)	4.64 (1.23)	0.952
Anti-PLA2R positive^3^	71 (75.5%)	35 (70.0%)	14 (77.8%)	22 (84.6%)	0.425
Anti-PLA2R titer level in those with positive anti-PLA2R level (RU/mL)	100.0 (32.0, 100.0)	100.0 (32.0, 100.0)	100.0 (32.0, 320.0)	82.0 (32.0, 100.0)	0.798
*Medications during study period*					
ACEi/ARB	36 (36.7%)	16 (30.8%)	7 (36.8%)	13 (48.1%)	0.345
Steroids	75 (76.5%)	42 (80.8%)	14 (73.7%)	19 (70.4%)	0.532
Immunosuppression	69 (70.4%)	34 (65.4%)	15 (78.9%)	20 (74.1%)	0.532
Diuretic	28 (28.6%)	11 (21.2%)	8 (42.1%)	9 (33.3%)	0.186
Hypolipidemic	60 (61.2%)	34 (65.4%)	8 (42.1%)	18 (66.7%)	0.171
Anticoagulant	56 (57.1%)	29 (55.8%)	9 (47.4%)	18 (66.7%)	0.426
Antiplatelet	40 (40.8%)	14 (26.9%)	13 (68.4%)^†^	13 (48.1%)	0.004*

^1^Data are presented as mean and SD

^2^Data are presented as median and IQR

^3^Data are presented as count and percentage

*Indicates a significant difference between the three groups

^†^Indicates a significant difference as compared with the CR group

^‡^Indicates a significant difference as compared with the PR group

*CR* complete remission, *PR* partial remission, *NR* no remission, *BMI* body mass index, *SBP* systolic blood pressure, *DBP* diastolic blood pressure, *SCr* serum creatinine, *BUN* blood urea nitrogen, *SUA* serum uric acid, *FBG* fasting blood glucose, *ALT* alanine aminotransferase, *AST* aspartate aminotransferase, *TC* total cholesterol, *TG* triglyceride, *LDL-C* low-density lipoprotein cholesterol; *HDL-C* high-density lipoprotein cholesterol, *PLA2R* phospholipase A2 receptor. *ACEi/ARB* angiotensin-converting enzyme inhibitor/ angiotensin II receptor blocker

### Renal pathology and immunology examination

A significantly higher proportion of patients in the CR and PR groups (71.2% and 73.7%, respectively) had no glomerular sclerosis compared to those in the NR group (37.0%) (both, *P* < 0.05). The proportions of tubular atrophy and interstitial fibrosis less than 5% of the CR group (both 88.5%) were significantly higher than those of the PR group (both 57.9%) and NR group (both 59.3%) (all, *P* = 0.003). No significant differences were observed between the three prognosis groups in the results of immunological examination (Table 2).

**Table 2:** Renal pathology and immunohistochemistry indices

	Total (*N* = 98)	CR (*N* = 52)	PR (*N* = 19)	NR (*N* = 27)	*P*-value
*Pathology examination*					
*Stage*					
I	22 (22.4%)	14 (26.9%)	3 (15.8%)	5 (18.5%)	
II	66 (67.3%)	35 (67.3%)	12 (63.2%)	19 (70.4%)	0.376
III + IV	10 (10.2%)	3 (5.8%)	4 (21.1%)	3 (11.1%)	
*Glomerular sclerosis*					
0	61 (62.2%)	37 (71.2%)	14 (73.7%)	10 (37.0%)^†‡^	
< 10%	15 (15.3%)	6 (11.5%)	1 (5.3%)	8 (29.6%)	
10–20%	15 (15.3%)	7 (13.5%)	3 (15.8%)	5 (18.5%)	0.004*
20–30%	4 (4.1%)	2 (3.8%)	1 (5.3%)	1 (3.7%)	
> 30%	3 (3.1%)	0 (0.0%)	0 (0.0%)	3 (11.1%)	
*Segmental glomerulosclerosis*					
0	86 (87.8%)	49 (94.2%)	14 (73.7%)	23 (85.2%)	
< 10%	7 (7.1%)	1 (1.9%)	4 (21.1%)	2 (7.4%)	
10–20%	3 (3.1%)	1 (1.9%)	1 (5.3%)	1 (3.7%)	0.085
20–30%	1 (1.0%)	0 (0.0%)	0 (0.0%)	1 (3.7%)	
> 30%	1 (1.0%)	1 (1.9%)	0 (0.0%)	0 (0.0%)	
*Tubular atrophy*					
< 5%	73 (74.5%)	46 (88.5%)	11 (57.9%)^†^	16 (59.3%)^†^	
5–24%	24 (24.5%)	6 (11.5%)	8 (42.1%)^†^	10 (37.0%)^†^	0.003*
25–50%	1 (1.0%)	0 (0.0%)	0 (0.0%)	1 (3.7%)	
*Interstitial fibrosis*					
< 5%	73 (74.5%)	46 (88.5%)	11 (57.9%)^†^	16 (59.3%)^†^	
5–24%	24 (24.5%)	6 (11.5%)	8 (42.1%)^†^	10 (37.0%)^†^	0.003*
25–50%	1 (1.0%)	0 (0.0%)	0 (0.0%)	1 (3.7%)	
*Mesangial proliferation*					
Grade 0	26 (26.5%)	14 (26.9%)	4 (21.1%)	8 (29.6%)	
Grade 1	63 (64.3%)	34 (65.4%)	13 (68.4%)	16 (59.3%)	0.920
Grade 2	9 (9.2%)	4 (7.7%)	2 (10.5%)	3 (11.1%)	
*Immunology examination*					
IgG	96 (98.0%)	50 (96.2%)	19 (100.0%)	27 (100.0%)	0.705
IgA	26 (26.5%)	10 (19.2%)	7 (36.8%)	9 (33.3%)	0.202
IgM	69 (70.4%)	34 (65.4%)	16 (84.2%)	19 (70.4%)	0.340
C3d	96 (98.0%)	50 (96.2%)	19 (100.0%)	27 (100.0%)	0.705
C4d	96 (98.0%)	50 (96.2%)	19 (100.0%)	27 (100.0%)	0.705
C1q	35 (35.7%)	14 (26.9%)	9 (47.4%)	12 (44.4%)	0.153

Data are presented as count and percentage

*Indicates a significant association between the corresponding variables and the three groups

^†^Indicates a significant difference as compared with the CR group

^‡^Indicates a significant difference as compared with the PR group

*CR* complete remission, *PR* partial remission, *NR* no remission

### UACR diurnal variation rate

**Fig. 1:**
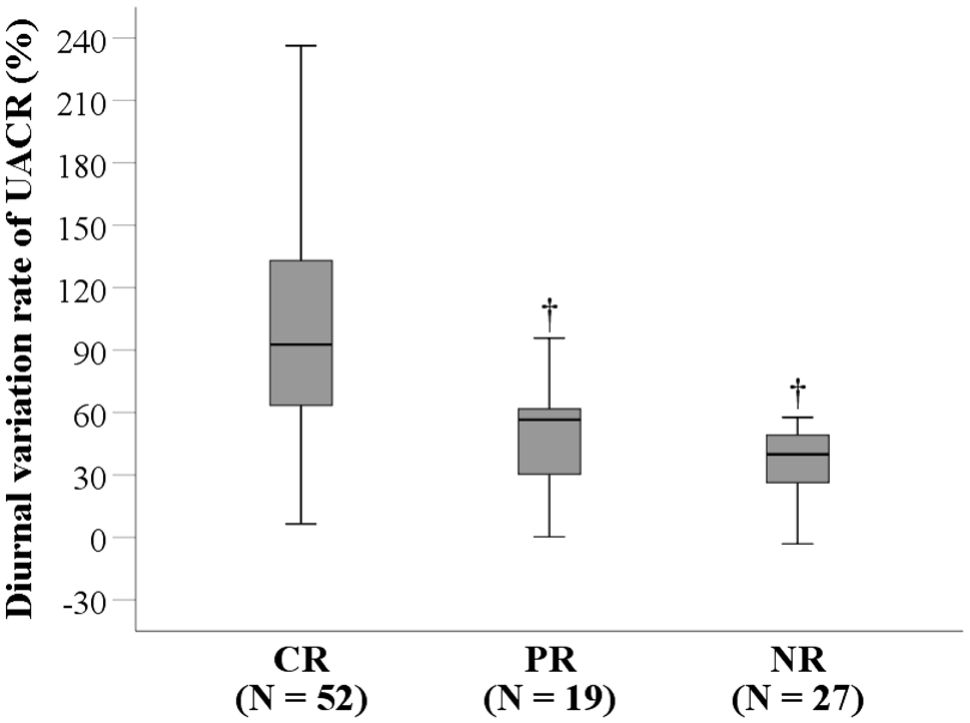
UACR diurnal variation rate. Patients in the CR, PR, and NR groups had median rates of 92.7% (IQR = 63.5–133.0), 56.6% (IQR = 29.2–63.6) and 39.9% (IQR = 22.3–50.1), respectively. ^†^Indicates a significant difference as compared with the CR group. *CR* complete remission, *PR* partial remission, *NR* no remission, *UACR* urine albumin-to-creatinine ratio

UACR_7:00_ and UACR_19:00_ values are listed in Supplementary Table 1. The median UACR diurnal variation rate was 59.4% (IQR = 37.4–103.0) for all the patients. Patients in the CR group had significantly higher UACR diurnal variation rates than those in the PR (92.7% vs. 56.6%) and NR (92.7% vs. 39.9%) groups (both *P* < 0.001)(Fig. 1).

### Predictors for NR

To find the independent predictors for NR, the variable in Tables 1 and 2 were analyzed using the univariable and multivariable logistic regression model, and the results are presented in Table 3. Results of the univariable logistic regression analysis present in the left side of Table 3 show that older age and higher 24-h urinary protein, BUN, and SUA significantly correlated with higher risk of NR, indicated by the odds of NR being increased with each unit increase in the above variables; patients with tubular atrophy, interstitial fibrosis, or glomerular sclerosis had significantly higher risk of NR than those without these conditions. Besides, higher UACR diurnal variation rates were correlated with significant lower risk of NR, the odds of NR decreased with each unit increase in UACR diurnal variation rate (OR = 0.97, *P* < 0.001).

**Table 3:** Univariable and multivariable logistic regression analysis for identify predictors for NR

	Odds ratio (95% CI)	*P* value	Adjusted odds ratio (95% CI)	*P* value
Age (years)	1.09 (1.03, 1.16)	0.002*	1.14 (1.03, 1.27)	0.014*
*Hypertension*				
Yes	2.19 (0.88, 5.46)	0.091		
No	Reference group			
*Nephrotic syndrome*				
Yes	3.35 (0.71, 15.76)	0.126		
No	Reference group			
*Tubular atrophy*				
≥ 5%	2.80 (1.07, 7.35)	0.037*		
< 5%	Reference group			
*Interstitial fibrosis*				
≥ 5%	2.80 (1.07, 7.35)	0.037*		
< 5%	Reference group			
*Glomerular sclerosis*				
Yes	4.33 (1.70, 11.06)	0.002*	11.15 (2.29, 54.17)	0.003*
No	Reference group		Reference group	
*ACEi/ARB*				
Yes	1.94 (0.78, 4.78)	0.151		
No	Reference group			
*24-h urinary protein (g/day)*	1.22 (1.03, 1.46)	0.022*	1.58 (1.13, 2.22)	0.008*
*SCr (μmol/L)*	1.03 (1.00, 1.06)	0.079		
*BUN (mmol/L)*	1.59 (1.19, 2.13)	0.002*	1.67 (1.07, 2.59)	0.023*
*SUA (μmol/L)*	1.005 (1.00, 1.01)	0.046*	1.01 (1.004, 1.03)	0.008*
*FBG (mmol/L)*	1.23 (0.90, 1.67)	0.192	2.26 (1.27, 4.03)	0.006*
*TG (mmol/L)*	1.17 (0.96, 1.41)	0.113		
*Platelet (× 10*^*9*^*/L)*	0.99 (0.99, 1.00)	0.103		
*Anti-PLA2R titer (positive to negative)*	2.133 (0.649, 7.009)	0.212		
*UACR diurnal variation rate (%)*	0.97 (0.95, 0.99)	< 0.001*	0.96 (0.94, 0.99)	0.001*

In univariable logistic regression models for binary outcomes of NR vs. CR or PR, variables with *p *values of crude odds ratios < 0.2 are listed on the left side. These variables were entered into the model selection process, and were summarized on the right side

^*^Indicates a significant association with clinical prognosis

*CR* complete remission, *PR* partial remission, *NR* no remission, *ACEi/ARB* angiotensin-converting enzyme inhibitor/ angiotensin II receptor blocker; *SCr* serum creatinine, *BUN* blood urea nitrogen, *SUA* serum uric acid, *FBG* fasting blood glucose, *TG* triglyceride, *UACR*, urine albumin-to-creatinine ratio

Multivariable logistic regression model was further conducted, and the results are presented in the right side of Table 3. Older age, higher 24-h urinary protein, higher BUN, and higher SUA correlated with higher risk of NR, with adjusted ORs (aORs) 1.14 (p = 0.014), 1.58 (*P* = 0.008), 1.67 (*P* = 0.023), and 1.01 (*P* = 0.008), respectively. Patients with glomerular sclerosis had significantly higher risk of NR than those without this condition, with an aOR of 11.15 (*P* = 0.003). The odds of NR decreased with each unit increase in UACR diurnal variation rate with an OR 0.96 (*P* = 0.001). FBG did not achieve significance in univariable analysis, but was significant in the final multivariable logistic regression model. Higher FBG correlated with higher risk of NR with aOR2.26 (*P* = 0.006).(Table 3).

**Fig. 2:**
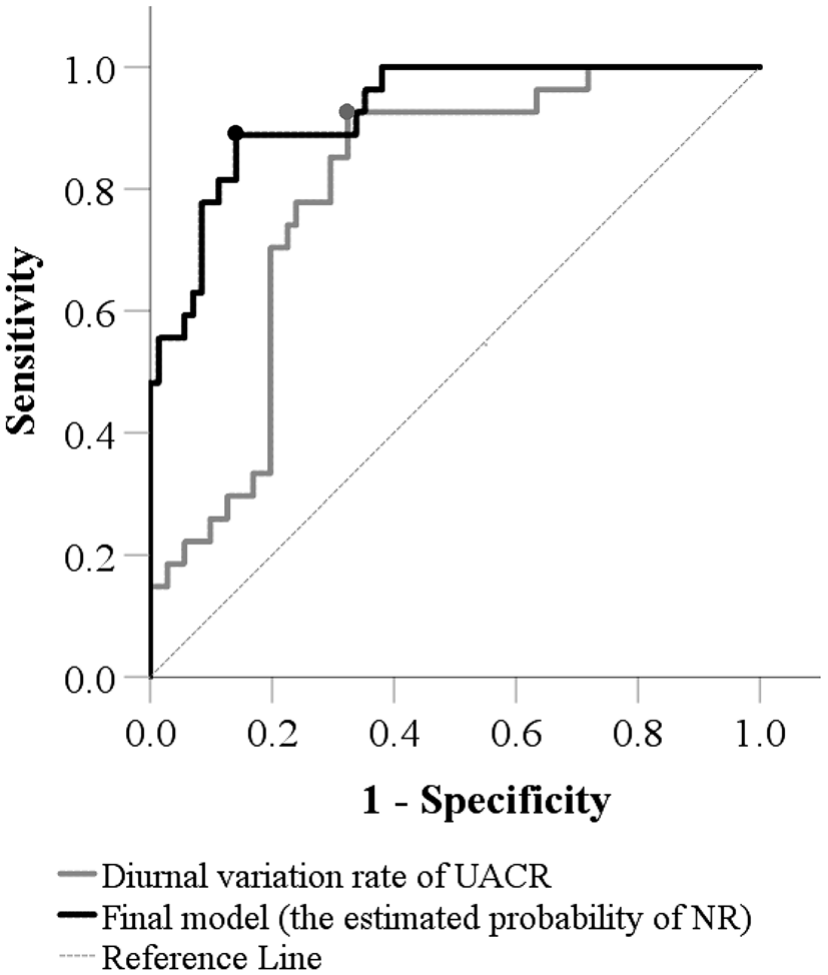
ROC analysis for the estimated probability of NR and UACR diurnal variation rate (%). The black and gray points are the optimal points determined by the maximum Youden’s index (sensitivity + specificity-1); on the two points, the estimated probability of NR and UACR diurnal variation rate (%) was 0.331 and 58.5%. *NR* no remission, *UACR* urine albumin-to-creatinine ratio

The predicted probability of NR can be calculated using the following formula developed based on the final multivariable logistic regression model: estimated probability of NR = exp(L)/(1 + exp(L)), where the linear predictor L =  – 22.038 + 0.134 × Age (years) + 0.457 × 24-h urinary protein + 0.511 × BUN + 0.014 × SUA + 2.411 if glomerular sclerosis + 0.816 × FBG-0.039 × UACR diurnal variation rate (%). ROC analysis showed that when using the estimated probability of NR to predict NR, the AUC was 0.927 (95% CI 0.876–0.979) and the optimal points determined by the maximum of Youden’s index was 0.331(Fig. 2). Using the optimal points on ROCs, patients with an estimated probability of NR over 0.331 were classified as NR, with sensitivity and specificity 0.889 and 0.859, NPV and PPV 0.953 and 0.706, and accuracy 0.867 (Table 4).

**Table 4:** Evaluation for UACR diurnal variation rate and final model of clinical prognosis of NR

Prediction criteria		NR (*N* = 27)	CR + PR (*N* = 71)	Sensitivity	Specificity	NPV	PPV	Accuracy
UACR diurnal variation rate	≤ 58.5%	25	23	0.926	0.676	0.960	0.521	0.745
	> 58.5%	2	48					
Predicted probability of the final model	≥ 0.331	24	10	0.889	0.859	0.953	0.706	0.867
	< 0.331	3	61					

*CR* complete remission, *PR* partial remission, *NR* no remission, *NPV* negative predictive value, *PPV* positive predictive value, *UACR* urine albumin-to-creatinine ratio

In addition, using the UACR diurnal variation rate only to predict NR, the AUC was 0.796 (95% CI 0.705–0.887) and the optimal points determined by the maximum of Youden’s index was 58.5% (Fig. 2). Patients with UACR diurnal variation rate 58.5% or lower were classified as NR, with sensitivity and specificity 0.926 and 0.676, NPV and PPV 0.960 and 0.521, and accuracy 0.745 (Table 4).

### Influence of UACR diurnal variation rate

**Fig. 3:**
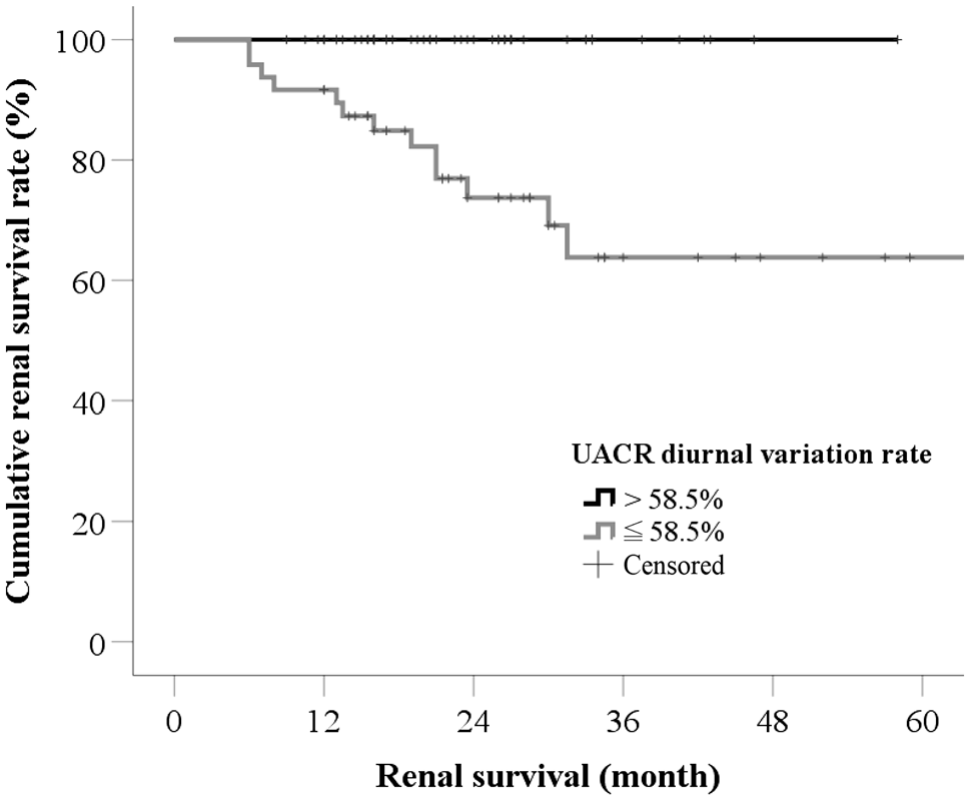
The cumulative rate of CR or PR. In patients with UACR diurnal variation rate > 58.5%, 48 patients achieved CR or PR with median time 6.0 months; and 33 patients with UACR diurnal variation rate ≤ 58.5% achieved CR or PR with median time 23.5 months. Log-rank test showed that patients with UACR diurnal variation rate > 58.5% had significantly higher cumulative rate of CR or PR than those with UACR diurnal variation rate ≤ 58.5% (*P* < 0.001). *CR* complete remission, *PR* partial remission, *NR* no remission, *UACR* urine albumin-to-creatinine ratio

**Fig. 4:**
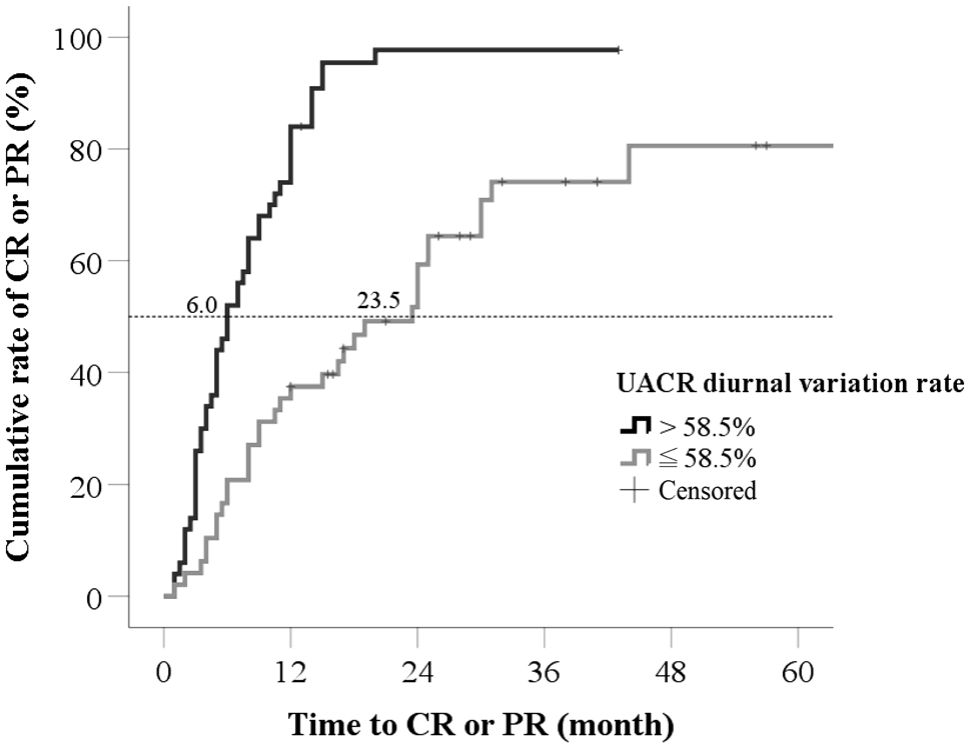
The cumulative renal survival rate. In those with UACR diurnal variation rates > 58.5%, composite renal outcomes did not occur in any patients, but did occur in 13 patients with UACR diurnal variation rate ≤ 58.5%. Log-rank test showed that patients with UACR diurnal variation rate > 58.5% had significantly higher renal survival rates than those with UACR diurnal variation rates of 58.5% (*P* < 0.001). UACR, urine albumin-to-creatinine ratio

Among the 98 IMN patients, 50 had UACR diurnal variation rates > 58.5%, and 48 had UACR diurnal variation rates ≤ 58.5%. The two groups had different long-term prognoses. In those with UACR diurnal variation rate > 58.5%, composite renal outcomes did not occur in any patient and 48 achieved CR or PR during the follow-up period (Fig. 3, *P* < 0.001). In those with UACR diurnal variation rate ≤ 58.5%, composite renal outcomes occurred in 13 patients, and 33 achieved CR or PR during the follow-up period. Patients with UACR diurnal variation rate > 58.5% needed median 6.0 months to achieve CR or PR, but those with UACR diurnal variation rate ≤ 58.5% needed 23.5 months. Those with UACR diurnal variation rate > 58.5% had significantly higher cumulative CR or PR rates than those with UACR diurnal variation rate ≤ 58.5% (Fig. 4, *P* < 0.001).

## Discussion

In the present study, the clinical application of UACR diurnal variation rate using spot urinary protein, a new marker for proteinuria grading was evaluated. We evaluated two methods that both including UACR diurnal variation rate to estimate the probability of NR; one is the final model that established based on the statistical results of comprehensive clinical data of patients with IMN, the other is using UACR diurnal variation rate only. Final model including age, 24-h urinary protein, BUN, SUA, glomerular sclerosis, FBG and UACR diurnal variation rate was developed from comprehensive assessments of IMN patients at baseline, using UACR diurnal variation rate, demographic and clinical data, blood and urinary biomarkers, and pathological characteristics. The sensitivity and specificity is 0.889 and 0.859, respectively, and accuracy is 0.867. Using the UACR diurnal variation rate alone as a simpler tool to predict NR, the sensitivity 0.926 is higher than the final model, and specificity 0.676 and accuracy 0.745 are both lower. Comprehensive assessment provides valuable information to help healthcare professionals make decisions, however, such assessment may not be user-friendly in day-to-day clinical practice. Seven variables selected statistically must be recorded for the final model, and the calculation is relatively complicated. To simplify the practice in busy clinical settings, the single-indicator UACR diurnal variation rate was also evaluated in the present study. Although the specificity and accuracy were lower than those in the final model, the higher sensitivity (0.926 vs. 0.889) indicated that UACR diurnal variation rate may be used more properly as a first-line screening tool for selecting patients who need complete assessments using the final model. However, regular visits and careful monitoring performed by the nephrologists is no doubt the best way to gain the largest benefit of the patients.

At presentation of IMN, age, proteinuria grade, and renal function are known as the predictors of risk assessment [16, 17]. Direct indicators of proteinuria grade and renal function included 24-h urinary protein, BUN, SUA, and UACR diurnal variation rate having significant associations between risk and NR was predictable. Interestingly, FBG, but not DM, gained sufficient weight to be incorporated into the final model. Most studies have evaluated the relationship between the two diseases, because IMN and DM both show renal function deterioration and are leading causes of ESRD [4, 18]. In addition, the coexisting factors become more frequent; IMN was found to be the most common glomerular disease in diabetic patients, and a high proportion of new-onset DM was observed in IMN patients [19–21]. More importantly, concurrent DM made IMN management more challenging, because glucocorticoids and immunosuppressants may interfere with glycemic management [6, 21]. However, the impact of DM on IMN outcomes is still elusive. Xie et al. [21] compared the remission rate of patients with or without DM and concluded that even though patients with concomitant DM experienced worse renal function deterioration, no significant differences were found in remission rates between the two groups. However, another study also conducted in China, but with a larger sample size, demonstrated that concomitant DM at IMN presentation is an independent risk factor for failure to achieve CR [22]. Results of the present study may reflect that FBG is a more sensitive indicator compared to DM, or just that the selection of statistical methods resulted in this finding, which needs further evaluation to clarify the different roles of FBG and DM in risk assessment for patients with IMN.

Hypertension, chronic kidney disease (CKD), DM, and monosymptomatic nocturnal enuresis are considered circadian-impacted renal disorders [23]. Renal function is an ultimate result of filtration, reabsorption and secretion, and all of these have been observed that have circadian rhythm [24]. Whether IMN severity or outcome is associated with kidney circadian rhythms is still uncertain. However, circadian variations of glomerular function have been observed in many studies. GFR reaches the maximum during the daytime, and decreases to the minimum in the middle of the night, and a similar trend is shown in the filtered load of water and sodium, urinary albumin, and β_2_-microglobulin excretion [10, 25, 26]. Results indicate that the time of specimen collection is an important factor for the accuracy of estimating glomerular function. Using data from multiple spot urinary values at different times within the day may overcome the bias from circadian variations. Because of the previous observations, we assumed that there may be some predictive value of kidney circadian rhythms for IMN and chose UACR diurnal variation rate to evaluate in the present study; UACR diurnal variation rate is considered as an alteration of 24-h urinary protein excretion for determining the degree of proteinuria. That is, a reflection or phenomenon of renal function deterioration, and may be associated to the final outcome of patients with IMN in addition to other renal disorders. UACR variation rate between 7:00 and 19:00 was chosen based on our preliminary data from 40 patients with IMN. Six time points were selected for evaluation: 7:00 hours (before breakfast), 9:00 hours (2 h after breakfast), 11:00 hours (before lunch), 13:00 hours (2 h after lunch), 17:00 hours (before dinner), and 19:00 hours (2 h after dinner). Not only the time of activity and inactivity (for humans, day and night, respectively), but diet was also considered because it is known to affect circadian rhythms of the kidneys and should be monitored [27]. UACR values between the six time points were significantly different (*P* < 0.05, data not shown), and the largest difference between the two time points was found between 7:00 and 19:00 and were thus chosen for further analysis in the present study. To our best knowledge, no attempt of applying UACR variation in addition to UACR diurnal variation rate for renal disease evaluation was done, and the preliminary findings may be extended to other renal or glomerular disorders in the future. Previous studies have found that in patients with nephrotic syndrome, proteinuria, blood pressure, and plasma sodium have circadian rhythm [28–30]; however, the mechanism that causes UACR variation is still unclear due to limited evidence. Several genes were also identified by genetic approaches including RNA-seq analysis and genome-wide association studies that were expressed in a circadian pattern in the kidney [31–33], but their direct impact on UACR is absent. Furthermore, knockout key circadian gene to disrupt renal clock activity probably does not lead to function impairment. In a doxycycline-inducible, nephron-specific knockout of *Bmal1* mouse model, no circadian pattern changes of urinary sodium, potassium and water excretion were observed. However, increased plasma creatinine was noted in the same animal model, indicating the possibility of impaired tubular function [34]. More evidences are required to construct a pathway in molecular level for the mechanism of UACR diurnal variation. To identify patients with higher risk of progression and who will benefit most from immunotherapy is clinically important to avoid unnecessary drug exposure because of the slow disease course of IMN and less than half of patients will progress to ESRD eventually [35, 36]. The KDIGO 2012 recommendations suggest postponing immunotherapy until at least after 6 months of observation under initial non-immunosuppressive agents, and only patients who fail to demonstrate significant improvement in proteinuria would be considered for immunotherapy [6]. However, delaying proper treatment may increase the risk of progression. Thus, timely identification of patients who need immunotherapy using reliable predictors is a critical step for treatment planning. Previous studies have indicated that patients with impaired renal function and severe proteinuria at baseline are more likely to progress to ESRD [37–40]; however, one study still demonstrated that serum albumin and urinary protein creatinine ratio used in current assessments indicated the opposite prediction: i.e., patients in an ethnically diverse cohort who reached ESRD had less severe nephrosis at baseline [41].This result is not surprising because IMN is a heterogeneous disease, including the pathogenesis mechanism, clinical presentation, and patient characteristics, which makes accurate prediction of outcomes difficult. Improvement of assessments used for diagnosis, treatment selection, monitoring of disease progression and therapeutic efficacy is worthy of extra effort and attention. In the present study, we used routine test variables for patients suspected of having renal disorder, but with slight modifications, i.e.. the UACR diurnal variation rate, which probably can be applied to real-world clinical practice more efficiently after verification of generalizability. As such, further studies are needed with larger samples, multiple geographic sites and ethnic groups, and longer observation periods.

Anti-PLA2R is considered as a key diagnostic biomarker of IMN [2, 6] and suggested the association between the presence of anti-PLA2R and clinical course [42, 43]. However, no significant difference in the presence of anti-PLA2R-positive rate or the titer of anti-PLA2R was found among the three groups in this study (*P* = 0.425 and 0.798, respectively). A meta-analysis indicated that compared to patients with the lowest titer of anti-PLA2R, the CR and spontaneous remission rate were significantly declined in IMN patients with the highest titer [44], indicating that the quantitative measures may be required for prognosis prediction purpose. However, in the present study, even when the titer of anti-PLA2R was incorporated into the univariable logistic regression model, no significant difference was found (odds ratio 2.133 with 95% CI 0.649–7.009, *P* = 0.212). The conflict results need more evaluation to clarify any difference between our cohort and those of previous studies.

The possible impact of treatment on nephrotic outcome was not analyzed in the present study, which should be noted as the limitation. The treatment was decided by the nephrologists according to KDIGO 2012 recommendations [6]. The data is too heterogenetic to be analyzed for the relation between treatment type and patient outcome. In addition, the treatment response should be determined after completion the treatment at least 6 months [6], and this data should be collected by a well-controlled clinical trial. Alternatively, a more practical definition of treatment failure should be found in the future for these kinds of investigation.

In conclusion, compared to 24-h urine, UACR diurnal variation is a simpler tool by which to predict nephrotic outcomes using spot urine protein testing, and by considering circadian rhythms of the kidneys. In addition, UACR diurnal variation rate provides more precise information than spot urinary testing alone in patients with IMN, while saving time and reducing the workload of healthcare professionals. UACR diurnal variation has higher sensitivity for more accurate identification of patients who should undergo further comprehensive assessment.

### Supplementary Information

Below is the link to the electronic supplementary material.

Supplementary file1 (DOCX 15 KB)

## Data Availability

All data generated or analyzed during this study are included in this article and its supplementary material files.
