# Supplementary material for: Urine albumin-to-creatinine ratio diurnal variation rate predicts outcomes in idiopathic membranous nephropathy
Source: Clin Exp Nephrol. 2024 Jan 19;28(5):409–20. doi: 10.1007/s10157-023-02444-9 (PMC11033241; doi:10.1007/s10157-023-02444-9)
Supplement: Supplementary file 1 — Supplementary file1 (DOCX 15 KB) [file 10157_2023_2444_MOESM1_ESM.docx]

Supplementary Table 1. UACR_7:00_ and UACR _19:00_ of the CR, PR and NR groups.

|  | **Total  (N = 98)** | **CR  (N=52)** | **PR  (N=19)** | **NR  (N=27)** | **P-value** |
| --- | --- | --- | --- | --- | --- |
| UACR_7:00_ | 2,560.3 (1,501.0, 4,971.1) | 2,400.8 (1,590.2, 3,485.9) | 3,678.5 (2,276.8, 7,103.6) | 2,517.1 (1,041.7, 6,139.0) | 0.107 |
| UACR_19:00_ | 4,637.1 (2,683.2, 7,030.7) | 4,088.6 (2,926.3, 6,612.4) | 5,833.1 (2,686.8, 8,414.5) | 4,843.0 (2,459.4, 8,033.2) | 0.649 |
